# A History of the Development of *Brucella* Vaccines

**DOI:** 10.1155/2013/743509

**Published:** 2013-06-03

**Authors:** Eric Daniel Avila-Calderón, Ahidé Lopez-Merino, Nammalwar Sriranganathan, Stephen M. Boyle, Araceli Contreras-Rodríguez

**Affiliations:** ^1^Departamento de Microbiología, Escuela Nacional de Ciencias Biológicas, Instituto Politécnico Nacional, Prolongación Carpio y Plan de Ayala s/n, Col. Sto. Tomás, 11340 México, DF, Mexico; ^2^Center for Molecular Medicine and Infectious Diseases, Virginia-Maryland Regional College of Veterinary Medicine, Virginia Tech, Blacksburg, VA 24060, USA

## Abstract

Brucellosis is a worldwide zoonosis affecting animal and human health. In the last several decades, much research has been performed to develop safer *Brucella* vaccines to control the disease mainly in animals. Till now, no effective human vaccine is available. The aim of this paper is to review and discuss the importance of methodologies used to develop *Brucella* vaccines in pursuing this challenge.

## 1. Introduction

Brucellosis is a zoonosis affecting approximately 500,000 people annually around the world. The disease remains endemic in many regions of the world including Latin America, Middle East, Africa, Asia, and the Mediterranean basin [1]. *Brucella *can be acquired by humans when they come in direct contact with fluid discharges from an infected animal, but in endemic regions people usually get infected through the consumption of unpasteurized dairy products mainly goat's milk and fresh soft cheese made out of unpasteurized milk. Isolation of *Brucella* is the gold standard to confirm infection; however, this is time-consuming and requires skilled technicians. Also, the handling of samples containing bacterial pathogen represents a high risk for laboratory personnel, for example, brucellosis is the most common bacterial laboratory-acquired infection worldwide [1, 2]. Therefore, brucellosis is generally diagnosed based on serological tests, in both animals and humans. However, human brucellosis is often misdiagnosed and underreported basically because the flu-like symptoms are protean and not specific. The treatment of human brucellosis requires the prolonged use of combination of antibiotics [3]. *Brucella* as facultative intracellular pathogen establishes an intimate relationship with the immune cells of the host. Through the subversion of the immune system, the pathogen is able to maintain a chronic infection that often makes treatment and diagnosis difficult. In the last decades, much research has been conducted in an attempt to develop safer and more effective *Brucella* vaccines for animals. There is no licensed vaccine for prevention of human brucellosis. A human vaccine would be useful to protect farmers, veterinarians, animal care workers, laboratory personnel, and general population living in endemic brucellosis areas [2]. The aim of this paper is to review and discuss the importance of the development of *Brucella* vaccines and some of the current methodologies used to pursue this goal.

## 2. Background

Research focused on the development of an ideal *Brucella* vaccine to prevent brucellosis in animals and humans has been sought since the beginning of twentieth century [4]. Live vaccines as well as inactivated ones have been developed; nevertheless, modified live vaccines have been shown to be superior protective immunogens as is the case with most diseases caused by facultative intracellular pathogens. 

The attenuated live vaccine *B. suis* strain 2 was extensively used in several animals species in China. This vaccine did not induce persistent antibodies. *B. abortus* strain 104-M was developed in China and used as vaccine in humans. This vaccine was reported to be stable in antigenic structure, virulence, and immunogenicity [4]. Immunity derived from the use of live attenuated vaccines tends to be cell mediated and long lasting. Also, as they are administered live, the organism is allowed to replicate within the host allowing *in vivo* gene expression, thus making them less expensive [5]. 

Two live attenuated vaccines strains were selected for their safety, stability, and immunogenicity for human protection. In 1952, *B. abortus* VA 19 was used in a mass epidemiological campaign in the former USSR, while in China *B. abortus* 104 M was tested in humans by intradermal, oral, and nasal routes [6]. 

Over the years, a wide variety of killed vaccines have been developed for protection against brucellosis. They have had limited acceptance and success. None have approached the protection levels afforded by the live attenuated vaccines. Examples of killed vaccines are *B. abortus *strain 45/20 and *B. melitensis *H38 [7]. *B. abortus *strain 45/20 was used in cattle and sheep, while *B. melitensis* H38 was tested in mice and cows. In addition to the lack of sufficient protection after challenge, killed vaccines such as strains 45/20 and H38 can induce persistent antibody titers. Killed vaccines for humans were first used in Malta in 1906 by Eyre, who vaccinated 51 soldiers [6]. 

Several antigenic fractions extracted from *Brucella* have been tested as a vaccine candidates, mainly in association with a variety of adjuvants; some of them included cell envelopes [8], outer membrane proteins [8, 9], insoluble residues of hot sodium dodecyl sulfate (SDS) extracts of cell envelopes (PG) [9], phenol insoluble fraction (PI) [10], soluble SDS extracts [11], *Brucella* soluble antigens (BASA) [12], periplasmic proteins and salt extractable proteins [13], chemically modified *Brucella* proteins [14], smooth and rough LPS [15], recombinant Cu-Zn superoxide dismutase (SOD), and synthetic peptides [16], among others. From all these fractions, only the phenol insoluble (PI) fraction has been used in humans [10, 17]. PI was an extract from the cell wall of *B. melitensis*. PI was applied subcutaneously in two doses of 1 mg with 15 hours interval. In humans, this vaccine showed low toxicity, hypersensitivity, and good immune response. The PI vaccine was used massively in 1967 in 800 laboratory workers who were often in contact with the bacteria. It was reported that all of them were protected except one who had immunodeficiency. The protection conferred by PI lasted around 18 to 24 months [18].

## 3. Current Vaccines for the Prevention of Animal Brucellosis

Vaccination is probably the most economic measure for control of brucellosis in endemic areas. Many countries have developed control measures for the eradication of the disease in livestock animal. These programs minimized the economic losses due to the abortion, infertility, and weak offspring and decreased milk production [19]. Presently, the vaccination programs are based on control of brucellosis mainly due to *B. melitensis* and *B. abortus *[20, 21].

Females are the most important targets for the vaccination programs as some vaccines cause severe tissue damage in male and lateral or vertical transmission occurs mainly via fluids associated with abortion or birth of infected calves or through shedding in milk. Vaccination by intramuscular or subcutaneous route is the most frequently used in livestock, but also the intraconjunctival route also has been used with good results [19]. 

Currently, only three live attenuated vaccines for the control of *B. abortus *infection in cattle are recommended: *B. abortus* 45/20, *B. abortus* strain 19 (S19), and *B. abortus* RB51 [5, 11, 22]. *B. abortus *strain 45/20 was isolated following twenty passages in guinea pigs; this rough strain is used only as heat-killed vaccine to avoid reversion to a virulent strain. Also this vaccine needs to be administrated with an adjuvant in adult cattle; it does not interfere with serological diagnosis and it is safer in pregnant animals but only has been tested in some countries [22].

Another licensed live smooth attenuated vaccine for control of bovine brucellosis is *B. abortus *S19. This strain was isolated in the early twentieth century and was naturally attenuated when a virulent culture of *B. abortus *was left at room temperature for one year [23]. *B. abortus* S19 has been effective for the control of brucellosis in adult bovines and prevents abortion as well as decreasing the prevalence in herds. However due to the smooth nature of the strain S19 and the strong antibody response against the O-side chain, it does not permit discrimination of infected from vaccinated animals. The competitive ELISA assay and radial immunodiffusion test have been used to permit differentiation between vaccinated or infected animals in field [24]. A low rate of abortion in livestock and significant reduction in milk production has been reported with *B. abortus* S19 vaccination [19, 22]. 

The live vaccine *B. abortus *RB51 is a spontaneous rough mutant obtained by subculturing the virulent strain *B. abortus* 2308 on medium containing rifampicin and penicillin [25]. Subsequently, it was found to contain an IS711 element disrupting the *wbo*A gene encoding a glycosyl transferase responsible for O-side chain synthesis [26]; hence it has a rough phenotype. In contrast to strain 45/20, *B. abortus* RB51 is very stable and it is currently used in many countries instead of *B. abortus* S19. Rough strain RB51 is less virulent and it does not induce a positive response in typical serological diagnostic test [5, 22, 25]. Vaccination of pregnant cows with strain RB51 can induce low levels of abortion (less than 0.2%); however it is safe at lower doses during pregnancy [27]. Vaccine strain RB51 can infect humans but it is less virulent than strain S19 [28]. 

Currently, the live vaccine *B. melitensis *Rev. 1 is used for the control of brucellosis in small ruminants. This strain was developed by Herzberg and Elberg in mid-1950s and retains the common characteristics of the *Brucella *species, but it is resistant to 2.5 *μ*g/mL streptomycin and susceptible to 5 IU penicillin G that allows differentiation from field strains [29]. Subcutaneous or conjunctival immunization with *B. melitensis* Rev. 1 confers adequate immunity in small ruminants. *B. melitensis* Rev. 1 induces a positive antibody response in serological tests in vaccinated animals. Also *B. melitensis* Rev. 1 can infect humans. The vaccination is recommended prior to the first gestation between 3 and 7 months of age to avoid abortion in pregnant animals [30]. As *B. melitensis* can be isolated from cattle, some scientists proposed use of the live attenuated vaccine *B. melitensis* Rev. 1 to control the disease in cattle [30].

Compared to the extensive research effort for developing new vaccines against *B. melitensis *or *B. abortus*, little research has been conducted to protect swine against *B. suis* [31]. Oral or intramuscular vaccination with strain RB51, killed *B. suis*, or purified O-polysaccharide was reported to be efficacious in protecting swine exposed to infected boars [29]. However, other studies have shown very little protection by strain RB51 in immunized swine against wild type challenge [19].

## 4. Brucellosis Eradication Programs

The general strategies proposed in 1998 by the WHO including Mediterranean Zoonoses Control Program to eradicate animal brucellosis were the following: (i) prevention of spread between animals and monitoring of brucellosis-free herds and zones, (ii) elimination of infected animals by test and slaughter programs to obtain brucellosis-free herds and regions, and (iii) vaccination to reduce the prevalence [32].

Due to the lack of a human vaccine against brucellosis, animal vaccination is a critical factor for the control and eradication of brucellosis in animals and humans. However this approach could be complemented with surveillance of livestock and/or elimination of infected animals as well as other critical control measures.

Regulatory programs for brucellosis are influenced by the prevalence of the disease in livestock or humans and economic considerations. The majority of programs for the eradication of animal brucellosis are aimed at reducing the prevalence that include test and removal programs, sanitization, and/or vaccination [19].

In general, sanitization programs are based on the education of the producers for elimination of contaminated material, decontamination, and other methods to avoid exposure and the dissemination of the disease agent. Test and removal programs are applicable in areas with high prevalence but not for eradication programs, and this should be stricter in surveillance herds of individual livestock [19]. However for developing countries, the elimination of the *Brucella*-infected animals is not affordable. 

In endemic regions where the prevalence of brucellosis was low and vaccination was stopped, outbreaks of human and animal brucellosis were reported [33].

In 2001, Brazil implemented control measures for eradication of bovine brucellosis, which included vaccination of the cows aged 3–8 months with *B. abortus* S19, accreditation of brucellosis-free herds, periodic surveillance, requirement of serological testing for movement or entry of livestock fairs, and compulsory slaughter of animals positive for brucellosis and permanent refresher training for accredited veterinarians [21]. It is clear that for the successful eradication and control programs for brucellosis in endemic areas, the government should be involved. They can raise the awareness of the risk of the disease, while also raising specific concern about infecting individual human subjects and the potential risk of horizontal transfer in the field. To circumvent these problems, the national veterinary services needed to maintain a certain level of competence as well as adopt a method to identify and register vaccinated animals. 

In the Middle East, brucellosis has been reported in almost all domestic animals, particularly cattle, sheep, and goats. However, there is a controversy on the best choice of the strategy for the brucellosis control. In some countries, the test and slaughter policy together with the vaccination of the young females was adopted; in others, particularly with regard to sheep and goats, mass vaccination was utilized. Regardless, vaccination was limited to cattle and small ruminants. In several countries, little is done to control the disease in humans because of lack of financial and technical facilities [34].

In the case of *B. suis*, dissemination in swine is primarily the result of *B. suis *biovar 1 and 3, and some cases of transmission of biovar 2 from wild pig to domestic swine have been reported. Due to the low pathogenicity of biovar 2 in humans, it is not considered to be a significant zoonotic threat and therefore likely not to have an adequate serologic surveillance [35, 36]. 

Although *B. ovis* rarely causes abortion in ewes, the infection manifests with testicular lesions and reduced infertility in rams [37]. As epididymal lesions in rams are not pathognomonic for the infection by *Brucella*, surveillance relies on serodiagnosis and isolation of the bacteria from infected animals [19, 38].

## 5. Subunit Vaccines against Brucellosis

As we mentioned earlier, the use of the live-attenuated vaccines against brucellosis represents a risk due to its potential ability to revert to virulence, cause abortion in pregnant animals, and be shed in milk, but also live strains could infect people coming into contact with the vaccine for example, farmers, abattoir workers, and veterinarians.

An ideal vaccine for use either in humans or in animals should meet the following criteria: should be effective and avirulent and induce long-lasting protection [39].

Subunit vaccines, like recombinant proteins, are promising vaccine candidates because they are less biohazardous, well defined, avirulent, noninfectious, and nonviable [40].

For the development of an effective vaccine against brucellosis, it is necessary to elicit an adequate immunological response (biased towards a Th1) and then choose the best antigen that induces protective immunity. It is well known that for an intracellular pathogen represented by *Brucella*, the production of IFN*γ*, TNF*α*, and IL-12 from the T helper cells (Th1 response), as well as CD8+ and CD4+ T lymphocytes, activated macrophages and dendritic cells are necessary for the control of the infection; whereas Th2 components have a minor role in the control of infection [21, 35, 36]. There appear to be three mechanisms of the adaptive immune response against brucellosis that are important: (1) production of IFN (produced by CD4+, CD8+, and *γδ* T cells) activates the bactericidal action of the macrophages to hamper the intracellular survival of *Brucella*; (2) the cytotoxic action of the CD8+ and *γδ* T cells kills infected macrophages; (3) Th1 antibody IgG2a isotypes opsonize the bacteria to facilitate effective phagocytosis [39, 41]. Subunit vaccines appear to be a promising option to develop safer vaccines because they are not able to regain virulence as opposed to live strains. However subunit vaccines also sometimes fail to elicit the magnitude of the immune response and/or induce protection comparable to live vaccines [39]. Since they tend to be poorly immunogenic stimulators, they require the coadministration of an adjuvant. Therefore the success of the subunit vaccine depends of the use of the substances endowed with immunomodulatory properties that control the selective induction of the appropriate type of antigenic-immune response. Moreover, the success of the subunit vaccine is associated with the route of administration [42].

### 5.1. Recombinant Proteins

Selecting the optimal antigens represents the cornerstone in vaccine design. Depending on the desired response, the antigenic proteins should contain appropriate epitopes to B-cell receptors and can be recognized by the T-cell receptor in a complex with MHC molecules [43].

Numerous subunit fractions from *Brucella* have been examined as recombinant proteins vaccines in mouse model, and some of these have shown protective efficacy [21, 39].

Several *Brucella *immunogenic antigens have been found in the outer membrane of this Gram-negative pathogen. Bacterial cell surface antigens are prime candidates as they represent the initial point of contact between the pathogen and its host [44].

For instance, the recombinant 31 kDa outer membrane protein (Omp31) in aluminum hydroxide or Incomplete Freud Adjuvant (IFA) induced significant levels of protection against *B. melitensis *challenge in the mouse model [45]. Also the purified recombinant lipoproteins Omp16 and Omp19 induce significant protection against oral or systemic *B. abortus *challenge when delivered by a parental or oral route without adjuvant, yet by a different immune mechanism. Also plant-made vaccines expressing Omp16 or Omp19 were able to induce significant protective immune response when administrated to mice by the oral route as purified proteins and within the crude leaf material of transgenic tobacco plants [40, 42].

The Omp25 (a peptidoglycan-layer protein) is an important *Brucella *virulence factor involved in survival; *B. melitensis*, *B. abortus*, and *B. ovis *Omp25 deletion mutants are attenuated in mice [46]. As a recombinant protein, Omp25 from *B. abortus *S19 induced protection against *B. abortus* 544 challenge in mice when it was administrated by intradermal or intraperitoneal routes [47].

In contrast, three peptides from the periplasmic protein Cu-Zn superoxide dismutase from *B. abortus *were assayed for induction of protection in mice; however only one was able to induce significant protection against *B. abortus *2308 [16].

Also the cytosolic proteins SurA and DnaK were evaluated in mice as purified recombinant proteins. Both proteins induced similar levels of protection against *B. abortus* (moderate levels compared with the live vaccine control) [48].

Combining several antigens in a vaccine formulation does not always induce higher levels of protection compared to the use of a single antigen. The immunization with DnaK and SurA did not show a synergistic effect compared with the vaccination of either antigen alone [48]. A similar effect was observed when Omp31 and *Brucella *lumazine synthase (BSL) were used together versus individually. However, chimeric formulation of Omp31-BSL augmented the protection achieved by either single antigen [49].

The need of the appropriate adjuvant is also important for obtaining protection in case of subunit vaccines. When recombinant periplasmic protein P39 was combined with CpG oligonucleotides, the protein induced a Th1 response and protection against *B. abortus* [50]. Recently recombinant Omp28 from *B. melitensis *adjuvanted with CpG conferred moderate levels of protection in mice against *B. abortus* 544 challenge [51].

### 5.2. Vectored Vaccines

Antigen delivery systems become necessary when antigens are not efficiently transported to the appropriate sites or presented to the immune system. For example, rapid degradation can result in weak or virtual lack of responses to otherwise immunogenic antigens [43].

BP26 is a 26 kDa periplasmic protein with an unknown function. Recently, a vectored vaccine has been developed based on protein BP26 or Omp16 and Omp31; that is, *E. coli* expressing *B. melitensis *BP26 induced lymphocyte proliferation, IFN-*γ* production, and protection in mice. On the other hand *E. coli *(K12) may be an ideal vaccine platform with natural adjuvant properties since it is nonpathogenic and can delivery antigens to antigen-presenting cells promoting cellular immune responses [52].

*Lactococcus lactis* expressing Cu-Zn SOD has also been used as a delivery system in mouse model and induced protection against a *B. abortus* challenge [53].

Semliki Forest Virus (SFV) was packed with RNA encoding the *B. abortus *Cu-Zn SOD and was able to induce protection in mice against *B. abortus* [54]. However, viral delivery systems do not always work, as in the case of the vaccinia virus delivering DNA encoding *B. abortus* GroEL (a chaperonin) or L7/L12 (ribosomal protein) was unable to induce a protective response against *B. abortus *challenge [55].

### 5.3. DNA Vaccines

DNA vaccines are able to induce both humoral and cellular immune responses. However, it is generally perceived that they induce less potent immune responses than protein vaccines [39]. Nevertheless, this may not be the case for brucellosis; for example, a DNA vaccine expressing Omp31 appears to elicit similar levels of protection as the recombinant protein combined with IFA [56]. Moreover, a BSL-DNA vaccine was more effective than the same recombinant protein against *B. abortus *challenge [57].

The use of the DNA vaccines resulted in the induction of a diverse immune response, which led to different levels of protection, which, in many cases, were not as high when compared with the commercial live attenuated *Brucella *vaccine [21].

Strategies to enhance the efficacy of DNA vaccines are constantly emerging to maximize immune responses. This is of particular importance in order to aid the transition of these vaccines into larger animal models and also humans [39].

One advantage of DNA vaccines is that multiple antigens can be expressed. A fusion of the antigens L7/L12 and Omp16 induced higher level of protection in mice than DNA vaccines expressing individual antigens [58]. Another study reported the fusion of L7/L12 and P39 genes (encoding ribosomal and periplasmic proteins, resp.) within a DNA vaccine that improved the protection compared with individual DNA vaccines encoding either alone [59].

In contrast, the coadministration of multiple plasmids expressing BSCP31 (31 kDa protein), Cu/Zn SOD, or L7/L12 strikingly enhanced the protection against *B. abortus *challenge compared with live vaccine *B. abortus* strain S19 [60].

Another strategy to improve brucellosis DNA vaccines is through the modulation of the immune response by the coexpression of cytokines as adjuvants [21]. When genes encoding IL-2 or IL-18 were fused to SOD and expressed in a single DNA vaccine, improved protection was observed compared to a SOD DNA vaccine alone [61, 62]. In contrast, when IL-15 or IL-12 genes were coadministrated on separate plasmids along with the multigenic vaccine containing the *bscp*31, *sod*, and L7/L12 genes, their efficacy was increased against *B. abortus* challenge compared to that observed with live vaccine S19 [63, 64].

### 5.4. Outer Membrane Vesicles: New Promising Vaccines against Brucellosis

Over 50 years ago, it was reported that Gram-negative bacteria shed outer membrane vesicles (OMVs) [65]. Production of these vesicles occurs spontaneously and during the normal growth of Gram-negative bacteria in many different environments including soil, biofilms, and enriched culture medium and during the infective process in the case of pathogens. As OMVs are shed from the outer membrane, their general composition has been characterized to contain outer membrane proteins, phosphoslipids, lipopolysaccharide (LPS), and some periplasmic compounds that are entrapped during the bulging of the outer membrane [66–69].

OMVs from the Gram-negative bacteria have been implicated in many processes including the release of virulence factors to the host as well as in the delivery of toxins, modulation of the immune system, trafficking of signaling molecules between bacterial cells, and biofilm formation [67–69]. Taking advantage of the immunomodulatory role of the OMVs from Gram-negative pathogens, the use of the OMVs as acellular vaccines against the OMVs-producing pathogens *in vivo *and *in vitro* has been tested [67, 70].

*Brucella* also release OMVs to the external milieu [71, 72]. Recently, the use of *Brucella* OMVs as a potential vaccine has been explored. Purified OMVs from both *B. melitensis *strains 16 M (smooth strain) and VTRM1 (rough lacking O-side chain) by differential centrifugation were used to immunize mice. When *Brucella* OMVs were administrated by an intramuscular route, OMVs from both strains induced similar levels of protection against virulent *B. melitensis *challenge compared with the live vaccine *B. melitensis* Rev. 1. In contrast, rough OMVs induced better levels of IgG2a antibodies than OMVs from smooth and live vaccine strain [73].

To improve the immune responses and subsequent protection by *Brucella *OMVs, Pluronic-85 was used as adjuvant. This adjuvant enhances the immune response and the protection against challenge with virulent *B. melitensis *strain compared to OMVs alone [74].

Compared to other subunit vaccines, OMVs may possess considerable advantages. OMVs are multicomplex antigens due to their numerous bacterial components and are composed of bacterial phospholipids that can act as natural bacterial adjuvants. Recently, it has been demonstrated that OMVs can interact with the eukaryotic host cell and enter by endocytic pathways; OMVs from *Brucella *are not the exception [75]. Therefore not only can membrane receptor dependent-pathways be induced, but also cytoplasmic receptors such as NOD (nucleotide binding and oligomerization receptor) or others could help trigger an immune response.

Compared to recombinant proteins, DNA vaccine, or vectored vaccines, OMVs could be less expensive in terms of production and purification. OMVs are purified from the *Brucella *culture medium by differential centrifugation and washing [73].

Because the genome sequences of many *Brucella* species are available, this makes it possible to genetically modify the content of OMVs using recombinant DNA technology, for example, overexpression of protective antigens that are expressed *in vivo*. In this way, it should be possible to enhance the efficacy of *Brucella* OMVs as has been reported by others [67, 68]. However, further research is essential to fully evaluate the benefits and risks of these types of acellular vaccines for the prevention of brucellosis in animals and especially humans.
